# Multiple Trait Covariance Association Test Identifies Gene Ontology Categories Associated with Chill Coma Recovery Time in *Drosophila melanogaster*

**DOI:** 10.1038/s41598-017-02281-3

**Published:** 2017-05-25

**Authors:** Izel Fourie Sørensen, Stefan M. Edwards, Palle Duun Rohde, Peter Sørensen

**Affiliations:** 1 Center for Quantitative Genetics and Genomics, Department of Molecular Biology and Genetics, Aarhus University, 8830 Tjele, Denmark; 2 The Roslin Institute and Royal (Dick) School of Veterinary Studies, The University of Edinburgh, Easter Bush, Midlothian, Scotland UK; 3 Centre for Integrative Sequencing, iSEQ, Aarhus University, 8000 Aarhus, Denmark; 4 iPSYCH, The Lundbeck Foundation Initiative for Integrative Psychiatric Research, 8000 Aarhus, Denmark

## Abstract

The genomic best linear unbiased prediction (GBLUP) model has proven to be useful for prediction of complex traits as well as estimation of population genetic parameters. Improved inference and prediction accuracy of GBLUP may be achieved by identifying genomic regions enriched for causal genetic variants. We aimed at searching for patterns in GBLUP-derived single-marker statistics, by including them in genetic marker set tests, that could reveal associations between a set of genetic markers (genomic feature) and a complex trait. GBLUP-derived set tests proved to be powerful for detecting genomic features, here defined by gene ontology (GO) terms, enriched for causal variants affecting a quantitative trait in a population with low degree of relatedness. Different set test approaches were compared using simulated data illustrating the impact of trait- and genomic feature-specific factors on detection power. We extended the most powerful single trait set test, covariance association test (CVAT), to a multiple trait setting. The multiple trait CVAT (MT-CVAT) identified functionally relevant GO categories associated with the quantitative trait, chill coma recovery time, in the unrelated, sequenced inbred lines of the *Drosophila melanogaster* Genetic Reference Panel.

## Introduction

The genomic best linear unbiased prediction (GBLUP) model has proven to be useful for estimation of population genetic parameters (e.g. heritability) as well as prediction of complex traits^1, 2^. GBLUP is a “black box” modelling approach fitting fixed and random effects simultaneous, utilizing the genetic relationship between individuals based on the correlation structure among genetic markers. Typically, GBLUP ignores prior biological information. Although models ignoring the underlying biology can serve as useful tools for prediction of genetic values or phenotypes, models utilizing known biological mechanisms provide a functional tool for testing our understanding of those mechanisms, and potentially improve inference and prediction accuracy.

It appears that markers associated with trait variation are not uniformly distributed throughout the genome, but enriched in genes that are connected in biological pathways^3–7^. Such knowledge could be utilized to build models that quantify the joint effect of a set of markers located in a genomic feature, i.e. genomic regions defined by e.g. genes, biological pathways, sequence annotation or other external evidence^8–10^. Improved inference and prediction accuray of GBLUP may be achieved by identifying genomic regions enriched for causal genetic variants.

The GBLUP approach can be modified in several ways to utilize genomic features as prior information. One approach is to extend the traditional GBLUP model to include additional genomic effects based on genetic markers located within a genomic feature^11–16^. Applying the genomic feature best linear unbiased prediction (GFBLUP) model to the *Drosophila* Genetic Reference Panel (DGRP)^17, 18^, we have previously demonstrated, that GFBLUP models can increase prediction accuracy for quantitative traits^15^. These results were further supported by simulation studies illustrating the impact of trait- and genomic feature-specific factors on prediction accuracy^15^. The GFBLUP model approach is, however, computationally intensive. An alternative approach is to search for patterns in GBLUP-derived single-marker statistics that can reveal associations between a genomic feature and a complex trait. We have previously evaluated a number of GBLUP-derived set tests on a binary outcome (i.e. disease trait) using high-density single nucleotide polymorphisms (SNPs) from genotyping arrays^19^. These GBLUP-derived set tests proved to be computationally fast and powerful compared to existing set test approaches^19^.

Here, we evaluated GBLUP-derived set tests on a quantitative trait as opposed to the binary outcome in the study of Rohde *et al*.^19^, and applied it to whole genome sequence data contrary to the genotypes derived from SNP arrays as previously shown^19^. Whole genome sequence data greatly exacerbate the true genomic signal to non-causal marker noise problem and may influence the power of set tests. Extending GBLUP-derived set tests could potentially increase detection power and contribute to a better understanding of complex traits’ underlying genetic architecture. First, multiple feature sets can be fitted in the model (e.g. a GFBLUP model), such as grouping markers based on their minor allele frequency^19, 20^ or prior QTL information^16^. By fitting multiple feature sets, genetic effects are estimated based on a mixture of normal distributions enabling further differential shrinkage of single marker effects across feature sets. Second, a multiple trait GBLUP model^21, 22^ can be fitted. This can potentially increase the accuracy of the total genomic value^21, 22^ and thereby the single marker effect, which in turn will lead to more accurate test statistics for genetic marker sets, thereby increasing detection power of the set test.

The aim of the study was to evaluate and compare genetic marker set tests derived from GBLUP on a quantitative trait using whole genome sequence data. Different set tests were evaluated and compared using simulated data generated from DGRP genotypes, focussing on factors specific to genomic features (e.g. the number, location and effect sizes of the true causal variants in the feature) that influence the power of set tests to detect genomic features affecting the trait phenotype. Furthermore, we investigated whether the results obtained using the GBLUP-derived set tests can be used to develop more accurate GFBLUP prediction models. Finally, we derived a multiple trait GBLUP set test (MT-CVAT) and used it to identify genomic features associated with a quantitative trait phenotype, chill coma recovery time (CCRT), in the unrelated, sequenced inbred lines of the DGRP.

## Methods

In the following a range of different GBLUP-derived set test approaches will be described in detail. The general procedure is to obtain single marker effects based on a standard GBLUP model, from which it is possible to compute and evaluate a test statistic for a set of genetic markers, measuring the degree of association between the genomic feature and the complex trait phenotype. This includes the statistical model and the underlying assumptions, test statistics for the set of genetic markers, and statistical procedures for assessing the statistical significance of the observed test statistic under a specific null hypothesis.

### Set test approach

The GBLUP-derived set test approach is based on two steps: First a standard linear mixed model is fitted, and then a test statistic for the marker set is computed.

### Linear mixed model

GBLUP is based on a linear mixed model including only one random genomic effect:

1 $${\bf{y}}={\bf{Xb}}+{\bf{Zg}}+{\bf{e}},$$

where **y** is the vector of phenotypic observations, **X** and **Z** are design matrices for the fixed and random effects, **b** is a vector of fixed effects, **g** is the vector of genomic values captured by all genetic markers, and **e** is the vector of residuals. The random genomic values and the residuals were assumed to be independent normally distributed values described as follows:$\,{\bf{g}} \sim {\rm{N}}({\bf{0}},{\bf{G}}{{\rm{\sigma }}}_{{\rm{g}}}^{2})\,$ and ${\bf{e}} \sim {\rm{N}}({\bf{0}},{\bf{I}}{{\rm{\sigma }}}_{{\rm{e}}}^{2})$. Thus, we assume that the observed phenotypes ${\bf{y}} \sim {\rm{N}}({\bf{Xb}},{\bf{V}})$ where ${\bf{V}}={\bf{ZGZ}}^{\prime} {{\rm{\sigma }}}_{{\rm{g}}}^{2}+{\bf{I}}{{\rm{\sigma }}}_{{\rm{e}}}^{2}$.

The additive genomic relationship matrix **G** is constructed^23^ using all genetic markers as follows: **G** = **WW′**/m, where **W** is the centered and scaled genotype matrix, and m is the total number of markers. Each column vector of **W** was calculated as follows: ${{\bf{w}}}_{{\bf{i}}}=\frac{{{\bf{a}}}_{{\bf{i}}}-2{{\rm{p}}}_{{\rm{i}}}}{\sqrt{2{{\rm{p}}}_{{\rm{i}}}(1-{{\rm{p}}}_{{\rm{i}}})}}$, where p_i_ is the minor allele frequency of the i^th^ genetic marker and **a**_**i**_ is the i^th^ column vector of the allele count matrix, **A** which contains the genotypes coded as 0, 1 or 2 counting the number of the minor allele.

### Single marker statistics

Single marker effects $\widehat{{\bf{s}}}$ can be computed from the predicted total genomic value $\hat{{\bf{g}}}={\bf{G}}{\hat{{\rm{\sigma }}}}_{{\bf{g}}}^{2}{{\bf{V}}}^{-1}({\bf{y}}-{\bf{X}}\hat{{\bf{b}}})$ obtained from the GBLUP model as:

2 $$\widehat{{\bf{s}}}={\bf{W}}^{\prime} {({\bf{WW}}^{\prime} )}^{-1}\widehat{{\bf{g}}},$$

and the (co)variance of the single marker effects can be computed as:

3 $$\widehat{{\rm{Var}}}(\widehat{{\bf{s}}})={\bf{W}}^{\prime} {({\bf{WW}}^{\prime} )}^{-1}\widehat{{\rm{Var}}}(\widehat{{\bf{g}}}){({\bf{WW}}^{\prime} )}^{-1}{\bf{W}}^{\prime} .$$

In this expression, the (co)variance of the predicted genomic value $\widehat{{\rm{Var}}}(\widehat{{\bf{g}}})={\bf{G}}{\widehat{{\rm{\sigma }}}}_{{\rm{g}}}^{2}+{{\bf{C}}}^{{\rm{gg}}}$ can be derived from the inverse of the coefficient matrix of the mixed model equations^24, 25^ for GBLUP where **C** ^gg^ is the part of this equation system that corresponds to the total genomic value.

Assessing association of individual markers is based on a single marker test statistic such as the t-statistic and a threshold for this statistic.

4 $${{\rm{t}}}_{{\widehat{{\rm{s}}}}_{{\rm{j}}}}=\frac{{\widehat{{\rm{s}}}}_{{\rm{j}}}}{\sqrt{{\rm{Var}}({\widehat{{\rm{s}}}}_{{\rm{j}}})}},$$

where ${\rm{Var}}({\widehat{{\rm{s}}}}_{{\rm{j}}})$ is the estimate of variance of the j’th element of $\widehat{{\bf{s}}}$ obtained from the j’th element of the diagonal of the (co)variance matrix of the single marker effects. Under the null hypothesis that ${\widehat{{\rm{s}}}}_{{\rm{j}}}=0$, it is assumed that ${{\rm{t}}}_{{\widehat{{\bf{s}}}}_{{\bf{j}}}}$ follows a t distribution with df_e_ residual degrees of freedom. The residual degrees of freedom df_e_ is computed as tr(**I** − **H**), which is equivalent to n − tr(**H**) where n is the total number of phenotypic observations and tr(**H**) represents the degrees of freedom occupied by the penalised fit (e.g. the linear mixed model fit). The hat matrix **H** transforms **y** into $\widehat{{\bf{y}}}$.

### Set tests for genomic features

The set test statistics for the feature set can be computed in a number of ways. Below is described four different approaches all derived from the GBLUP model.

The **first set test statistic** is the covariance association test (CVAT)^19^, which considers the covariance between the total genomic effect for all markers $(\hat{{\bf{g}}}={\sum }_{{\rm{i}}=1}^{{\rm{m}}}{{\bf{w}}}_{{\rm{i}}}{\hat{{\rm{s}}}}_{{\rm{i}}})$ and the genomic effect for the feature $({\hat{{\bf{g}}}}_{{\rm{f}}}={\sum }_{{\rm{i}}=1}^{{{\rm{m}}}_{{\rm{f}}}}{{\bf{w}}}_{{\rm{i}}}{\hat{{\rm{s}}}}_{{\rm{i}}})$:

5 $${{\rm{T}}}_{{\rm{CVAT}}}=\hat{{\bf{g}}}^{\prime} {\hat{{\bf{g}}}}_{{\rm{f}}}=({\hat{{\bf{g}}}}_{{\rm{f}}}^{^{\prime} }+{\hat{{\bf{g}}}}_{{\rm{r}}}^{^{\prime} }){\hat{{\bf{g}}}}_{{\rm{f}}}={\hat{{\bf{g}}}}_{{\rm{f}}}^{^{\prime} }{\hat{{\bf{g}}}}_{{\rm{f}}}+{\hat{{\bf{g}}}}_{{\rm{r}}}^{^{\prime} }{\hat{{\bf{g}}}}_{{\rm{f}}}.$$

In this expression ${\widehat{{\bf{g}}}}_{{\rm{r}}}={\sum }_{{\rm{i}}=1}^{{{\rm{m}}}_{{\rm{r}}}}{{\bf{w}}}_{{\rm{i}}}{\widehat{{\rm{s}}}}_{{\rm{i}}}$ is the genomic effect for the remaining set of markers. The number of markers in feature and in the remaining set of markers is given by m_f_ and m_r_ respectively.

The distribution of this set test statistic under the competitive null hypothesis (genomic feature comprises randomly sampled markers) is unknown and an empirical distribution is required. An empirical distribution for the competitive null hypothesis can be obtained by sampling m_f_ columns in **W** at random.

The **second set test statistic** considered is a commonly used score based approach. It is derived from the first derivative of the likelihood as is Rao’s score test^26^. A key difference compared to Rao’s score test is that only the quadratic term in the first derivative form the basis of this test statistic^27–29^ from an argument that this is the only part that involves the data^30^. The score based approach used here is thus equivalent to the sequence kernel association test (SKAT)^28^. The score statistic can therefore be written as:

6 $${{\rm{T}}}_{{\rm{Score}}}=\frac{1}{2}({\bf{y}}-{\bf{Xb}})^{\prime} {{\bf{V}}}^{-1}{{\bf{G}}}_{{\rm{f}}}{{\bf{V}}}^{-1}({\bf{y}}-{\bf{Xb}}),$$

where the fixed effects **b** and the phenotypic covariance matrix **V** are estimated under a null model. The purpose of the null model is to adjust for environmental non-genetic factors, and for genetic factors not part of the genomic feature, including population structure. Several alternative null models can be used in the score test approach. If the GBLUP model is used as the null model the genomic effects can either be defined as ${\bf{g}} \sim {\rm{N}}(0,{\bf{G}}{{\rm{\sigma }}}_{{\rm{g}}}^{2})$ or alternatively $\,{\bf{g}} \sim {\rm{N}}(0,{{\bf{G}}}_{{\rm{r}}}{{\rm{\sigma }}}_{{\rm{r}}}^{2})$. In the first case the genomic relationship matrix is computed using all genetic markers and therefore the null model needs only to be fitted once. In the latter case, it is computed using only the genetic markers not included in the genomic feature which requires us to fit a different null model for each genomic feature. The set test statistic for the score approach can be re-written as:

7 $${{\rm{T}}}_{{\rm{Score}}}=\frac{1}{2}\widehat{{\bf{e}}}^{\prime} {\bf{Z}}{{\bf{G}}}_{{\rm{f}}}{\bf{Z}}^{\prime} \widehat{{\bf{e}}}=\frac{1}{2}\widehat{{\bf{e}}}^{\prime} {\bf{Z}}\frac{{{\bf{W}}}_{{\rm{f}}}{{\bf{W}}}_{{\rm{f}}}^{^{\prime} }}{{{\rm{m}}}_{{\rm{f}}}}{\bf{Z}}^{\prime} \widehat{{\bf{e}}},$$

where $\widehat{{\bf{e}}}={\widehat{{\bf{V}}}}^{-1}({\bf{y}}-{\bf{X}}\widehat{{\bf{b}}})$. The empirical distribution of the score set test statistic under the competitive null hypothesis is obtained by randomly sampling m_f_ columns in **W**. It is also possible to derive an approximate distribution using the Satterthwaite’s procedure of moment matching to approximate the null distribution of T_Score_ by a Gamma distribution^29^. The two parameters in the approximate distribution are calculated by matching the first and second moments (mean and variance) with those of the score set test statistic.

The **third test statistic** is based on the sum of the test statistic for all genetic markers belonging to the same genomic feature such as:

8 $${{\rm{T}}}_{{\rm{sum}}}=\sum _{{\rm{i}}=1}^{{{\rm{m}}}_{{\rm{f}}}}{{\rm{t}}}_{{\rm{i}}}^{2},$$

where t_i_ represents the i’th single variant test statistic, e.g. marker effects ($\widehat{s}$) or t-statistics. The distribution of this test statistic under the null hypothesis (associated markers are picked at random from the total number of tested genetic markers) is unknown and an empirical distribution is required. In this study both $\widehat{s}$ and the t-statistic in equation 4 were used to compute T_sum_.

The **fourth set test statistic** is based on counting the number of genetic markers in the feature that are associated with the trait phenotype and is computed as:

9 $${{\rm{T}}}_{{\rm{count}}}=\,\sum _{{\rm{i}}=1}^{{{\rm{m}}}_{{\rm{f}}}}{\rm{I}}({{\rm{t}}}_{{\rm{i}}} > {{\rm{t}}}_{{\rm{0}}}),$$

where m_f_ is the number of markers in the feature, t_i_ is the i’th single marker test statistic (e.g. t-statistic), t_0_ is an arbitrary chosen threshold for the single marker test statistics, and I is an indicator function that takes the value one if the argument (${\rm{abs}}({{\rm{t}}}_{{\rm{i}}}) > {{\rm{t}}}_{0}$) is satisfied. Under the null hypothesis (i.e. individually associated markers are distributed randomly, thus, the number of associated markers within a feature is indifferent compared to a random set of markers) it is assumed that ${{\rm{T}}}_{{\rm{count}}} \sim {\rm{Hyper}}({\rm{m}},{{\rm{m}}}_{{\rm{a}}},{{\rm{m}}}_{{\rm{f}}})$ is a realization from a hypergeometric distribution with parameters m (total number of genetic marker tested), m_a_ (total number of associated genetic markers amongst all markers) and m_f_ (total number of genetic markers in the feature). Alternatively, the statistical significance of the T_count_ statistic can be assessed using a *χ* ^2^ test for independence^31^ or by obtaining an empirical distribution under a specific null hypothesis.

### Extensions to GBLUP-derived CVAT

The CVAT is a flexible set test approach which can be extended in a number of ways facilitating further investigation of the underlying genetic architecture of complex traits. E.g. it can be decomposed at different levels of a hierarchy of gene sets, genes and markers; it can be derived from a model with multiple genetic components; or it can be derived from multiple trait models.

**First**, the CVAT statistic can be decomposed at different levels of a hierarchical genomic feature classification scheme, such as decomposing the covariance between the total genomic value and the genomic value defined by a genomic feature at the pathway level (e.g. group of genes) into the contribution from individual genes $({\widehat{{\bf{g}}}}_{{\rm{f}}}={\sum }_{{\rm{i}}=1}^{{{\rm{n}}}_{{\rm{genes}}}}{\widehat{{\bf{g}}}}_{{{\rm{f}}}_{{\rm{i}}}})$ to the covariance test statistics and even single markers $({\widehat{{\bf{g}}}}_{{{\rm{f}}}_{{\rm{i}}}}={\sum }_{{\rm{j}}=1}^{{{\rm{m}}}_{{{\rm{f}}}_{{\rm{i}}}}}{{\bf{w}}}_{{\rm{j}}}{\widehat{{\rm{s}}}}_{{\rm{j}}})$ within a gene. The number of SNPs ${{\rm{m}}}_{{{\rm{f}}}_{{\rm{i}}}}$ located within genes varies (due to gene size etc.) and therefore partitioned covariance test statistics at the gene level are presented “per SNP”.

**Second**, the CVAT statistic can be derived from a GFBLUP model with multiple genetic components^14–16, 19^. The total genomic values in the GBLUP model are assumed to be drawn from the same distribution ${\bf{g}} \sim {\rm{N}}(0,{\bf{G}}{{\rm{\sigma }}}_{{\rm{g}}}^{2})$. It is, however, very likely that the genomic values come from a mixture of distributions, e.g. groups of genetic markers having different effects based on their minor allele frequency (MAF)^20^ or genetic markers known a priori to have large effects (e.g. discovered in previous GWAS). Such prior information can be used by fitting multiple genetic components in the linear mixed model:

10 $${\bf{y}}={\bf{Xb}}+\sum _{{\rm{i}}=1}^{{{\rm{n}}}_{{\rm{f}}}}{\bf{Z}}{{\bf{g}}}_{{\rm{i}}}+{\bf{e}}.$$

The notation is similar to the GBLUP model presented above except **g**_i_ is the vector of genetic values captured by the i’th genetic marker set. The random genetic effects and residuals were assumed to be independent and distributed as ${{\bf{g}}}_{{\rm{i}}} \sim {\rm{N}}(0,{{\bf{G}}}_{{\rm{i}}}{{\rm{\sigma }}}_{{{\rm{g}}}_{{\rm{i}}}}^{2})$, and ${\bf{e}} \sim {\rm{N}}(0,{\bf{I}}{{\rm{\sigma }}}_{{\rm{e}}}^{2})$ where ${{\bf{G}}}_{{\rm{i}}}={{\bf{W}}}_{{\rm{i}}}^{^{\prime} }{{\bf{W}}}_{{\rm{i}}}/{{\rm{m}}}_{{\rm{i}}}$ is the additive genomic relationship matrix for the i’th genetic marker set. The single marker effects derived from the GFBLUP model are computed as: ${\hat{{\bf{s}}}}_{{\rm{i}}}={{\bf{W}}}_{{\rm{i}}}^{^{\prime} }{({{\bf{W}}}_{{\rm{i}}}{{\bf{W}}}_{{\rm{i}}}^{^{\prime} })}^{-1}{\hat{{\bf{g}}}}_{{\rm{i}}}$, thus $\widehat{{\bf{s}}}=[{\widehat{{\bf{s}}}}_{1}^{^{\prime} }\ldots {\widehat{{\bf{s}}}}_{{{\rm{n}}}_{{\rm{f}}}}^{^{\prime} }]$.

**Third**, the CVAT statistic can be derived from a multiple trait GBLUP model (or GFBLUP model)^21, 22^. This can be important if we have records on correlated traits, for example a high heritability trait (or a trait with many observations) correlated with a low heritability trait (or a trait with few observations). In such a situation using a multiple trait model is likely to increase the accuray of the predicted total genetic value and single marker effects for the low heritability trait which in turn will increase the power of the set test. This becomes highly relevant for borrowing information across traits or same trait recorded in different breeds or study populations. The linear mixed model for multiple traits (2 traits in this example) can be expressed as:

11 $$[\begin{array}{c}{{\bf{y}}}_{1}\\ {{\bf{y}}}_{2}\end{array}]=[\begin{array}{c}{{\bf{X}}}_{1}{{\bf{b}}}_{1}\\ {{\bf{X}}}_{2}{{\bf{b}}}_{2}\end{array}]+[\begin{array}{c}{{\bf{Z}}}_{1}{{\bf{g}}}_{1}\\ {{\bf{Z}}}_{2}{{\bf{g}}}_{2}\end{array}]+[\begin{array}{c}{{\bf{e}}}_{1}\\ {{\bf{e}}}_{2}\end{array}].$$

The notation is similar to the GBLUP model presented above except that **y**_1_ and **y**_2_ are vectors of phenotypes for trait 1 and 2, respectively. **X**_1_ and **X**_2_ are design matrices for the fixed effects and **b**_1_ and **b**_2_ are the vectors of these fixed effects. **Z**_1_ and **Z**_2_ are design matrices for the random effects, **g**_1_ and **g**_2_ are vectors of total genetic values and **e**_1_ and **e**_2_ are vectors of residuals for trait 1 and 2.

The random genetic effects, $${\bf{g}}=[\begin{array}{c}{{\bf{g}}}_{1}\\ {{\bf{g}}}_{2}\end{array}]$$, and residuals, $${\bf{e}}=[\begin{array}{c}{{\bf{e}}}_{1}\\ {{\bf{e}}}_{2}\end{array}]$$, were assumed to be independent and distributed as $${\bf{g}} \sim {\rm{N}}(0,{\bf{G}}\otimes [\begin{array}{cc}{{\rm{\sigma }}}_{{{\rm{g}}}_{11}}^{2} & {{\rm{\sigma }}}_{{{\rm{g}}}_{12}}^{2}\\ {{\rm{\sigma }}}_{{{\rm{g}}}_{21}}^{2} & {{\rm{\sigma }}}_{{{\rm{g}}}_{22}}^{2}\end{array}])$$, and $${\bf{e}} \sim {\rm{N}}(0,{\bf{I}}\otimes [\begin{array}{cc}{{\rm{\sigma }}}_{{{\rm{e}}}_{11}}^{2} & {{\rm{\sigma }}}_{{{\rm{e}}}_{12}}^{2}\\ {{\rm{\sigma }}}_{{{\rm{e}}}_{21}}^{2} & {{\rm{\sigma }}}_{{{\rm{e}}}_{22}}^{2}\end{array}])$$. Furthermore, T_CVAT_ can be used to identify features associated with the covariance between total genetic values in different traits expressed as:

12 $${{\rm{T}}}_{{\rm{CVAT}}}={\widehat{{\bf{g}}}}_{1}^{^{\prime} }{\widehat{{\bf{g}}}}_{{{\rm{f}}}_{2}}={\widehat{{\bf{g}}}}_{{{\rm{f}}}_{1}}^{^{\prime} }{\widehat{{\bf{g}}}}_{{{\rm{f}}}_{2}}+{\widehat{{\bf{g}}}}_{{{\rm{r}}}_{1}}^{^{\prime} }{\widehat{{\bf{g}}}}_{{{\rm{f}}}_{2}},$$

which consider the covariance between the total genomic effect for all markers $({\widehat{{\bf{g}}}}_{1}={\sum }_{{\rm{i}}=1}^{{\rm{m}}}{{\bf{w}}}_{{\rm{i}}}{\widehat{{\rm{s}}}}_{{1}_{{\rm{i}}}})$ of trait 1 (or trait 2) and the genomic effect for a feature $({\hat{{\bf{g}}}}_{{{\rm{f}}}_{2}}={\sum }_{{\rm{i}}=1}^{{{\rm{m}}}_{{\rm{f}}}}{{\bf{w}}}_{{\rm{i}}}{\hat{{\rm{s}}}}_{{2}_{{\rm{i}}}})$ of trait 2 (or trait 1).

### Fitting linear models and estimation of variance components

Estimates of the variance components (i.e. $\,{\widehat{{\rm{\sigma }}}}_{{\rm{g}}}^{2},{\widehat{{\rm{\sigma }}}}_{{{\rm{g}}}_{1}}^{2},{\widehat{{\rm{\sigma }}}}_{{{\rm{g}}}_{2}}^{2},{\widehat{{\rm{\sigma }}}}_{{{\rm{g}}}_{12}}^{2}\,{\rm{and}}\,{\widehat{{\rm{\sigma }}}}_{{\rm{e}}}^{2}$) defined in the models described above were obtained using an average information restricted maximum likelihood (AI-REML) procedure^32, 33^ as implemented in the software DMU. In this procedure, matrices were not full rank due to centering of the observed genotypes, which necessitated a generalized inverse of the genomic relationship matrices.

### Testing for association between a genomic feature and a phenotype

The test for association was based on a competitive null hypothesis, i.e. that the degree of association of the feature set was the same as that of a random marker set^27, 34^.

A null hypothesis is only competitive if the parameters influencing the test statistic are identical to the alternative hypothesis. Thus, there must be an equal number of markers for the random set and the true set, and the correlation structure among markers (due to linkage disequilibrium) should be retained. The empirical distribution of the test statistics was therefore obtained using the circular permutation procedure as described in Cabrera *et al*.^35^. The genome was considered to be circular, ordered from chromosome 2 L to chromosome X and restarting again at chromosome 2 L. Then the complete set of observed test statistics are permuted by rotation with respect to their genomic locations, i.e. a random number between 1 and the total number of SNPs is drawn, and the observed test statistic for the first SNP in the genome rotates to that of the random number-th SNP and all other test statistics rotate to the same degree to the corresponding SNPs. Thus, SNPs retain the same original order but, at each permutation, gain new random test statistics. This uncouples any associations between SNPs and the genomic feature, while retaining similar patterns of the correlation structure among test statistics. A new set test statistic was then computed based on the original position of the genomic features. The permutation was repeated 10,000 times for each set in the feature class, and empirical p-values were obtained through one-tailed tests of the proportion of randomly sampled test statistics larger than that observed.

### Implementation

The GBLUP-derived set test approaches described above were implemented in the R package qgg, which is available at http://psoerensen.github.io/qgg/. This includes fitting a series of linear mixed models, estimating variance components using methods such as REML, computing the test statistic for the set of genetic markers, and testing the statistical significance of the observed test statistic under a specific null hypothesis. Example scripts and data sets are provided for illustrating the GBLUP model derived set test approaches. For specific experimental design with replicated phenotypes within line such as DGRP it is more efficient to use the AI-REML procedure^32, 33^ implemented in DMU^32^. The AI-REML function in the qgg package provides an R interface to the DMU which can be downloaded from http://dmu.agrsci.dk/DMU/. The CVAT approach can also be derived from the REML procedures implemented in existing software packages commonly used in genomics such as GCTA^36^, LDAK^37^, DISSECT^38^ and MTG2^39^.

### Simulation study comparing set test approaches

To compare the different set test approaches described above, and to investigate different factors that might influence the power to detect causal sets of SNPs, a series of phenotypic simulations were established. The factors varied in the simulations should imitate different genetic architectures and included genomic heritability (h^2^), proportion of genomic variance explained by causal SNPs in the genomic feature (${{\rm{h}}}_{{\rm{f}}}^{2}$), proportion of non-causal SNPs in the genetic marker set defined by the genomic feature (*dilution*), genome distribution of causal SNPs (*causal model*, i.e. whether the causal SNPs were distributed in the genome randomly or clustered in groups) and the number of phenotypic records for each genotype (N_rep_). For each data set and replicate we estimated variance components for the GBLUP and GFBLUP models using AI-REML and applied the different set tests (not including the extensions to CVAT).

### Simulated data

The simulations were based on the real genotype DGRP data set of 205 lines. Genotypes were originally obtained from whole genome sequences using an integrative genotyping procedure^18^. All simulations were based on segregating biallelic single nucleotide polymorphisms (SNPs) with minor allele frequencies ≥0.05 and for which the Phred scaled variant quality was greater than 500 and the genotype call rate was ≥0.8, resulting in a total of 1,725,755 SNPs.

#### Causal sets

In all scenarios, there were 1,000 causal SNPs, which were divided into two subsets. The first subset, *C*_1_, contained 100 SNPs and was used as the causal SNP set in the genomic feature that explained 10%, 20%, 30%, or 50% of the genomic variance. The second subset, *C*_2_, contained 900 SNPs and explained the remaining genomic variance. To mimic relevant genetic scenarios, the genome distribution of the causal SNPs in the genomic feature was simulated using two different causal models: a *random* and a *cluster* model. The *cluster* model simulates the situation in which multiple causal SNPs occur in a limited number of genes, whereas in the random model single causal SNPs occur in a larger number of genes. The main difference is that the genomic variance is associated with a smaller genome region in the cluster model compared to the random model. For the clustered causal model, the 100 causal SNPs in *C*_1_ were chosen from 20 randomly selected genome regions spanning 50 SNPs each, and the remaining 900 SNPs in *C*_2_ were randomly selected from the complete SNP set (excluding the SNPs in *C*_1_). For the random causal model, the SNPs in *C*_1_ and *C*_2_ were randomly selected from the complete SNP set. To investigate the effects of non-causal SNPs within the causal sets, we added an increasing number of non-causal SNPs (100, 200, …, 1,900, 2,000), to the causal set *C*_1_, in a process referred to as *dilution*. To determine the false-positive rate, 50 non-causal SNP sets of varying sizes (10 sets each containing 0.1 k, 0.5 k, 1 k, 5 k or 10 k SNPs) were sampled, none of which were contained in the causal sets of SNPs.

#### Phenotypes

Phenotypes were simulated using the following linear model: $\,{\bf{y}}={\bf{Z}}{{\bf{g}}}_{1}+{\bf{Z}}{{\bf{g}}}_{2}+{\bf{e}}$, where ${{\bf{g}}}_{1} \sim {\rm{N}}(0,{{\bf{G}}}_{1}{{\rm{\sigma }}}_{{\rm{g}}1}^{2})$, ${{\bf{g}}}_{2} \sim {\rm{N}}(0,{{\bf{G}}}_{2}{{\rm{\sigma }}}_{{\rm{g}}2}^{2})$, and ${\bf{e}} \sim {\rm{N}}(0,{\bf{I}}{{\rm{\sigma }}}_{{\rm{e}}}^{2})$. **G**_1_ and **G**_2_ are the genomic relationship matrices for causal SNPs in *C*_1_ and *C*_2_, respectively. **Z** is a design matrix linking DGRP lines to individual phenotypes. The total phenotypic variance ${{\rm{\sigma }}}_{{\rm{P}}}^{2}={{\rm{\sigma }}}_{{\rm{g}}1}^{2}+{{\rm{\sigma }}}_{{\rm{g}}2}^{2}+{{\rm{\sigma }}}_{{\rm{e}}}^{2}$ was 100 in all scenarios. We simulated data with additive genomic heritabilities $({{\rm{h}}}^{2}=\frac{{{\rm{\sigma }}}_{{\rm{g}}1}^{2}+{{\rm{\sigma }}}_{{\rm{g}}2}^{2}}{{{\rm{\sigma }}}_{{\rm{g}}1}^{2}+{{\rm{\sigma }}}_{{\rm{g}}2}^{2}+{{\rm{\sigma }}}_{{\rm{e}}}^{2}})$ of 0.1, 0.3, or 0.5, to analyse scenarios with low to intermediate heritabilities, reflecting those observed in the real data. To analyse scenarios with non-uniform SNP effects, the proportion of additive genomic variance explained by the causal SNPs in *C*_1_ $({{\rm{h}}}_{{\rm{f}}}^{2}=\frac{{{\rm{\sigma }}}_{{\rm{g}}1}^{2}}{{{\rm{\sigma }}}_{{\rm{g}}1}^{2}+{{\rm{\sigma }}}_{{\rm{g}}2}^{2}})$ was varied across scenarios: 0.1, 0.2, 0.3, or 0.5. These parameters were investigated for N_rep_ of 5, 10, and 50. Increasing the number of replicates per line decreases the variance of the phenotypic value for each line. Combining these factors yielded a total of 1,440 individual simulated data sets [3 (N_rep_) × 3 (h^2^) × 4 (${{\rm{h}}}_{{\rm{f}}}^{2}$) × 2 (causal model) × 20 (dilution)]. For each possible combination of factors 50 independent data sets were obtained.

### Assessing the power of set test statistics

To measure the performance of the different test statistics we used the F_1_ score:

13 $${{\rm{F}}}_{1}=2\frac{{\rm{p}}\cdot {\rm{r}}}{{\rm{p}}+{\rm{r}}},$$

where ${\rm{p}}={\rm{TP}}/({\rm{TP}}+{\rm{FP}})$ is the precision and ${\rm{r}}={\rm{TP}}/({\rm{TP}}+{\rm{FN}})\,$ is the recall. The F_1_ score is the harmonic mean of precision and recall^40^. The recall *r* is the proportion of true positives (TP) that are correctly identified, i.e. the ratio between the number of identified causal sets and the number of sets that should have been identified, thus, the sum of TP and false negatives (FN). Contrary, the precision p is the proportion of positives that truly are positives, i.e. the proportion of true causal sets of all sets identified, thus, the sum of TP and false positives (FP). The F_1_ score can take values between 0 and 1, with maximum performance at the value of 1. The F_1_ score was calculated for each test staticstic under each combination of factors, using a p-value cut-off of 0.05 for a positive detection of a genomic feature.

### Comparing set test results with the predictive ability of the GFBLUP model

We investigated whether the results obtained using the GBLUP derived set tests can be used to develop more accurate GFBLUP prediction models. The GFBLUP model is an extension of the traditional GBLUP model, where an additional genomic effect (defined by the genomic feature) is included in the linear mixed model^15^. The predictive ability of the GFBLUP model was assessed using a cross validation procedure^15^. In the GFBLUP model the total genomic value is ${\widehat{{\bf{g}}}}_{{\rm{total}}}=\widehat{{\bf{f}}}+\widehat{{\bf{r}}}$, where $\widehat{{\bf{f}}}$ is a vector of genomic values captured by genetic markers linked to the genomic feature of interest, $\widehat{{\bf{r}}}$ is a vector of genomic values captured by genetic markers outside the genomic feature. In the cross validation procedure, we estimated genomic parameters using the phenotypes from the DGRP lines in the training data (90% of the lines) and predicted the total genomic value of DGRP lines in the validation data (10% of the lines). We then calculated Spearman correlations between the total genomic values predicted with or without the observed phenotypes set to missing. For the simulated data and for the observed DGRP data we defined 50 cross training (validation) data subsets and applied these to each genomic feature. For each genomic feature, the predictive ability was defined as the average correlation of the 50 cross validations. For comparing the GBLUP-derived set tests with the predictive ability of the GFBLUP model, we calculated the Spearman rank based correlation between the level of significance of the set test statistic and the predictive ability.

### CVAT (and its extensions) exemplified on CCRT

We applied the GBLUP-derived CVAT on CCRT measured in the DGRP. The CVAT test statistic was chosen based on its good performance in the simulation studies (see first section of Results). Individual genes and gene ontology (GO) terms defined genetic marker sets (genomic features) for which T_CVAT_ was computed. The relationship between this test statistic and the predictive ability of incorporating these GO terms as features in the GFBLUP model^15^ was considered.

### DGRP data

#### *Drosophila* lines

The phenotypic and genotypic data originate from the *Drosophila melanogaster* Genetic Reference Panel (DGRP)^17, 18^. All data can be accessed via the website: http://dgrp2.gnets.ncsu.edu/. The DGRP consists of 205 inbred lines obtained by 20 generations of full-sib mating from the offspring of single wild-caught females collected from the Raleigh, NC, USA population, and which have full genome sequence data available^17, 18^. All flies were reared under standard culture conditions (cornmeal-molasses-agar-medium, 25°C, 60–75% relative humidity, 12-hr light-dark cycle). The DGRP is polymorphic for common inversions and *Wolbachia pipientis* infection status^18^. These factors were included in the models described below as fixed effects.

#### Quantitative trait phenotype

Chill coma recovery time (CCRT) for 159 DGRP lines was measured by transferring three to seven day old flies without anesthesia to empty vials, and placing them on ice for three hours. Flies were transferred to room temperature, and the time it took for each individual to right itself and stand on its legs was recorded^41^. There were two replicates of ~50 flies/sex/line (total *N* = 32,231; female *N* = 16,170; male *N* = 16,061).

#### Genotypes

Genotypes were obtained from whole genome sequences using an integrative genotyping procedure^18^. All analyses were based on segregating biallelic single nucleotide polymorphisms (SNPs) with minor allele frequencies ≥0.05 and for which the Phred scaled variant quality was greater than 500 and the genotype call rate was ≥0.8, for a total of 1,725,755 SNPs distributed on six chromosome arms (*2L*, *2R*, *3L*, *3R*, *4* and *X*).

#### Genomic features

Genomic features were defined at gene-level and GO level. Genes grouped according to a specific GO term were considered a genomic feature. Genes were linked to the ‘Biological Processes’ (BP), ‘Molecular Function’ (MF), and ‘Cellular Component’ (CC) GO terms^42^ using the BioConductor package ‘org.Dm.eg.db’ v. 2.14^43^. Only GO terms with at least 10 directly evidenced genes were used in the analyses. SNPs were mapped to FlyBase genes using the v5.49 annotations of the *D*. *melanogaster* reference genome^17, 18, 44^. Only the 963,235 SNPs located within genes (i.e. within open reading frames) were used for the genomic feature. In total the markers were annotated to 10,517 genes and 1,117 GO terms. A total of 1,725,755 markers were used in all analyses, and the number of markers linked to a single GO term ranged from 23–163,938.

### Single and multiple trait CVAT

We applied CVAT to the CCRT data, and considered CCRT in males and females as two different, but correlated traits. The multiple trait CVAT analysis was based on phenotypic records of the quantitative trait adjusted for relevant factors using the following multi-trait linear mixed model:

14 $$[\begin{array}{c}{{\bf{y}}}_{1}\\ {{\bf{y}}}_{2}\end{array}]=[\begin{array}{c}{{\bf{X}}}_{1}{{\bf{b}}}_{1}\\ {{\bf{X}}}_{2}{{\bf{b}}}_{2}\end{array}]+[\begin{array}{c}{{\bf{Z}}}_{1}{{\bf{g}}}_{1}\\ {{\bf{Z}}}_{2}{{\bf{g}}}_{2}\end{array}]+[\begin{array}{c}{{\bf{Q}}}_{1}{{\bf{l}}}_{1}\\ {\bf{0}}\end{array}]+[\begin{array}{c}{\bf{0}}\\ {{\bf{Q}}}_{2}{{\bf{l}}}_{2}\end{array}]+[\begin{array}{c}{{\bf{e}}}_{1}\\ {{\bf{e}}}_{2}\end{array}],$$

**y**_1_ and **y**_2_ are vectors of phenotypes for trait 1 (males) and 2 (females), **X**_1_ and **X**_2_ are design matrices for fixed effects of inversion karyotypes and *Wolbachia* infection status and **b**_1_ and **b**_2_ are the vectors of these fixed effects. **Z**_1_ and **Z**_2_ are design matrices linking observations to genomic values, **g**_1_ and **g**_2_ are vectors of total genetic values. **Q**_1_ and **Q**_2_ are design matrices for replicate within line effects, **l**_1_ and **l**_2_ the vectors of replicate within line effects, and **e**_1_ and **e**_2_ are vectors of residuals for trait 1 and 2.

The random genetic effects,$$\,{\bf{g}}=[\begin{array}{c}{{\bf{g}}}_{1}\\ {{\bf{g}}}_{2}\end{array}]$$, line effects, $${\bf{l}}=[\begin{array}{c}{{\bf{l}}}_{1}\\ {{\bf{l}}}_{2}\end{array}]$$ and residuals, $${\bf{e}}=[\begin{array}{c}{{\bf{e}}}_{1}\\ {{\bf{e}}}_{2}\end{array}]$$ were assumed to be independent and distributed as $${\bf{g}} \sim {\rm{N}}(0,{\bf{G}}\otimes [\begin{array}{cc}{{\rm{\sigma }}}_{{{\rm{g}}}_{11}}^{2} & {{\rm{\sigma }}}_{{{\rm{g}}}_{12}}^{2}\\ {{\rm{\sigma }}}_{{{\rm{g}}}_{21}}^{2} & {{\rm{\sigma }}}_{{{\rm{g}}}_{22}}^{2}\end{array}]),\,{{\bf{l}}}_{1} \sim {\rm{N}}(0,{{\bf{I}}}_{{{\rm{l}}}_{1}}{{\rm{\sigma }}}_{{{\rm{l}}}_{1}}^{2}),\,{{\bf{l}}}_{2} \sim {\rm{N}}(0,{{\bf{I}}}_{{{\rm{l}}}_{2}}{\sigma }_{{{\rm{l}}}_{2}}^{2})\,{\rm{and}}\,{\bf{e}} \sim {\rm{N}}(0,{\bf{I}}\otimes [\begin{array}{cc}{{\rm{\sigma }}}_{{{\rm{e}}}_{11}}^{2} & {{\rm{\sigma }}}_{{{\rm{e}}}_{12}}^{2}\\ {{\rm{\sigma }}}_{{{\rm{e}}}_{21}}^{2} & {{\rm{\sigma }}}_{{{\rm{e}}}_{22}}^{2}\end{array}])$$. Since the phenotypes for males and females were recorded in different environments, we assume that ${{\rm{\sigma }}}_{{{\rm{e}}}_{12}}^{2}={{\rm{\sigma }}}_{{{\rm{e}}}_{21}}^{2}=0$.

The CVAT test statistic, T_CVAT_, was computed using the vectors of total genomic values in males and females, **g**_1_ and **g**_2_ from the multiple trait analyses. The within trait CVAT test statistics were computed as ${{\rm{T}}}_{{{\rm{CVAT}}}_{{\rm{M}}}}={\widehat{{\bf{g}}}}_{1}^{^{\prime} }{\widehat{{\bf{g}}}}_{{{\rm{f}}}_{1}}$ for males (trait 1) and ${{\rm{T}}}_{{{\rm{CVAT}}}_{{\rm{F}}}}={\widehat{{\bf{g}}}}_{2}^{^{\prime} }{\widehat{{\bf{g}}}}_{{{\rm{f}}}_{2}}$ for females (trait 2). The across trait CVAT test statistics were computed as ${{\rm{T}}}_{{{\rm{CVAT}}}_{{\rm{MF}}}}={\widehat{{\bf{g}}}}_{1}^{^{\prime} }{\widehat{{\bf{g}}}}_{{{\rm{f}}}_{2}}\,{\rm{or}}\,{{\rm{T}}}_{{{\rm{CVAT}}}_{{\rm{FM}}}}={\widehat{{\bf{g}}}}_{2}^{^{\prime} }{\widehat{{\bf{g}}}}_{{{\rm{f}}}_{1}}$ which consider the covariance between the total genomic effect for all markers $({\widehat{{\bf{g}}}}_{1}={\sum }_{{\rm{i}}=1}^{{\rm{m}}}{{\bf{w}}}_{{\rm{i}}}{\widehat{{\rm{s}}}}_{{{\rm{1}}}_{{\rm{i}}}})$ of trait 1 (or trait 2) and the genomic effect for a feature $({\widehat{{\bf{g}}}}_{{{\rm{f}}}_{2}}={\sum }_{{\rm{i}}=1}^{{{\rm{m}}}_{{\rm{f}}}}{{\bf{w}}}_{{\rm{i}}}{\widehat{{\rm{s}}}}_{{2}_{{\rm{i}}}})$ of trait 2 (or trait 1).

The single trait CVAT was done by analysing phenotypes for males and females separately using the same fixed and random factors as in the multiple trait model presented above.

An empirical distribution of these test statistics was based on a competitive null hypothesis using the permutation procedure described earlier. Two competive null hypotheses were used to test if the observed test statistics of the genomic feature differs from the test statistics obtained by randomly sampling genetic markers from (a) exclusively genic regions or (b) the whole genome (i.e. genic and intergenic regions). Thus empirical distributions were obtained by either sampling genetic markers randomly from gene regions or the whole genome.

## Results

### Comparison of set test statistics on simulated data

#### Comparison of power for set test statistics

**Figure 1:**
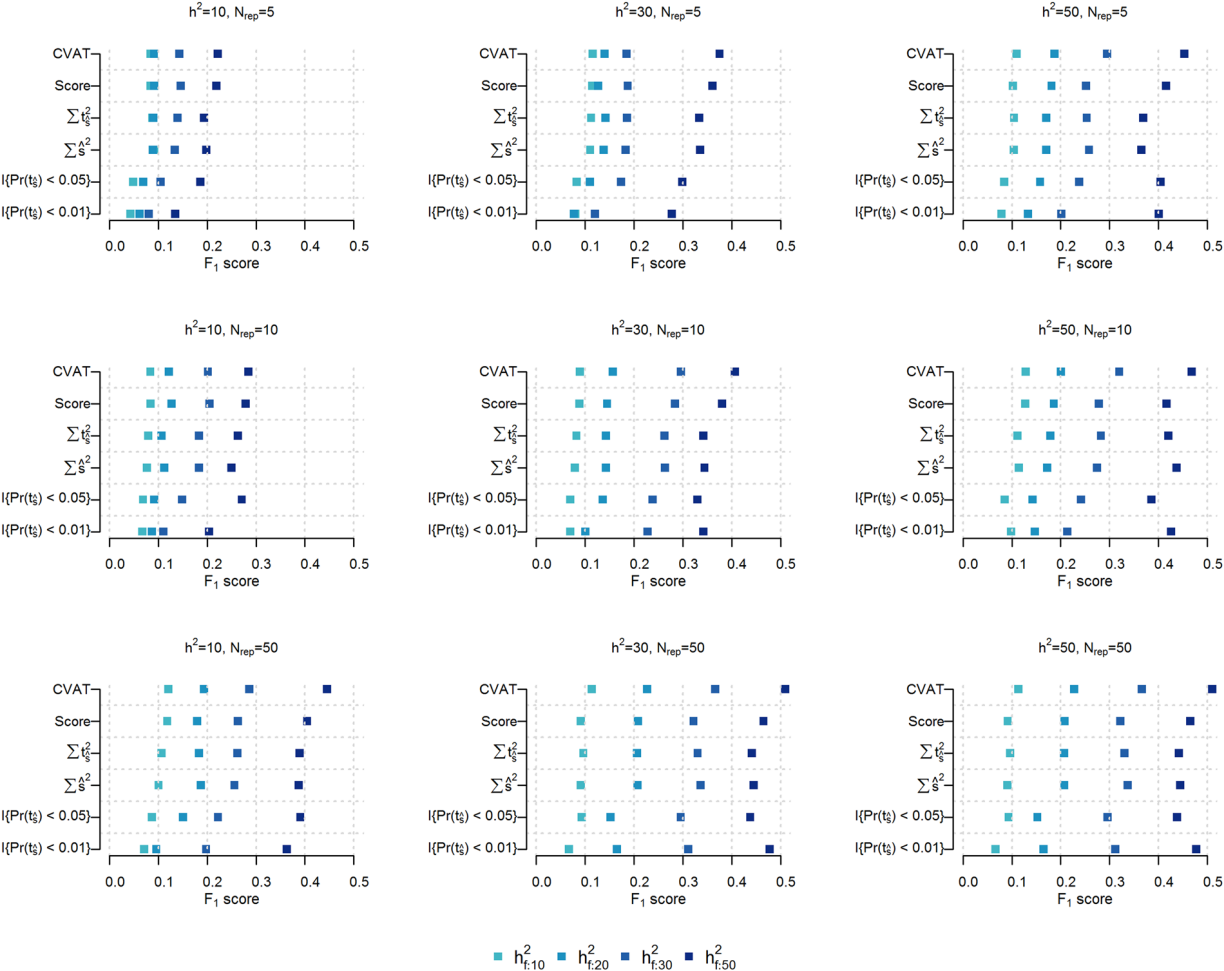
Comparison of detection power between set test statistics. The F_1_ score (x-axis) was used to measure the performance of the GBLUP derived set test statistics (y-axis), i.e. T_CVAT_ (CVAT), T_Score_ (Score), T_Sum_ using single marker effects $({\sum \widehat{{\rm{s}}}}^{2})$ or using single marker t-statistics $(\sum {{\rm{t}}}_{\widehat{{\rm{s}}}}^{2})$, and T_Count_ with a threshold of p-value < 0.05 $({\rm{I}}\{{\rm{\Pr }}({{\rm{t}}}_{\hat{{\rm{s}}}}) < 0.05\})$ and p-value < 0.01 $({\rm{I}}\{{\rm{\Pr }}({{\rm{t}}}_{\hat{{\rm{s}}}}) < 0.01\})$. The F_1_ score was calculated using the average set test statistic results over a dilution range of adding 0 to 2000 non-causal SNPs to the *C*_1_ causal set. P-value cut-off for the set test statistic was 0.05. Each panel represent a different combination of genomic heritability (h^2^) and number of replicates within lines (N_rep_), whereas ${{\rm{h}}}_{{\rm{f}}}^{2}$ is visualized by the colour gradient. Results are for the scenarios with three different levels of genomic heritability (h^2^ = 0.1, 0.3 or 0.5, columns left to right), four different levels of proportion of genomic variance explained by the causal markers in the genomic feature (${{\rm{h}}}_{{\rm{f}}}^{2}$ = 0.1, 0.2, 0.3 or 0.5, light to dark colour), and three different levels of number of replicates within lines (N_rep_ = 5, 10, or 50, rows top to bottom). Causal sets, including SNPs in feature (*C*_1_) and not in feature (*C*_2_), consisted of SNPs randomly selected from the complete SNP set (random causal model).

The covariance association test, T_CVAT_, was generally more powerful (i.e. highest F_1_ score) than other set test statistics, across all scenarios (Fig. 1) under the random model. The figure displays estimated power for different set test statistics across 3 different trait heritabilities (h^2^), three number of replicates (N_rep_), and 4 levels of proportion of additive genetic variance explained by causal SNPs (${{\rm{h}}}_{{\rm{f}}}^{2}$). The F_1_ score was calculated for the average of each set test result over a dilution range of 0 to 2,000 non-causal SNPs added to the *C*_1_ causal set. The superior performance of CVAT becomes more pronounced as the genomic heritability increases (left to right column of Fig. 1), genomic variance explained by feature increases (darker colour of points in Fig. 1), and number of replicates increase (top to bottom row of Fig. 1). Slightly less power was observed for the score based test statistic T_Score_ followed closely by the set test statistic T_Sum_ based on sums of single marker test statistics (marker effects $\widehat{{\rm{s}}}$ or t-statistics). This trend is observed across all scenarios. All of the aforementioned set test statistics mostly outperform the count based set test statistic T_Count_ at p-value cut-offs of 0.05 and 0.01. However, when the feature explains 50% of the genomic variance (i.e. ${{\rm{h}}}_{{\rm{f}}}^{2}$ = 0.5) the power of T_Count_ (using a stringent single marker p-value cut-off (p < 0.01)) improves, as heritability and number of replicates increase, such that it’s power reaches levels comparable to the score based set test statistic (Fig. 1).

#### Relationship between set test statistics

**Figure 2:**
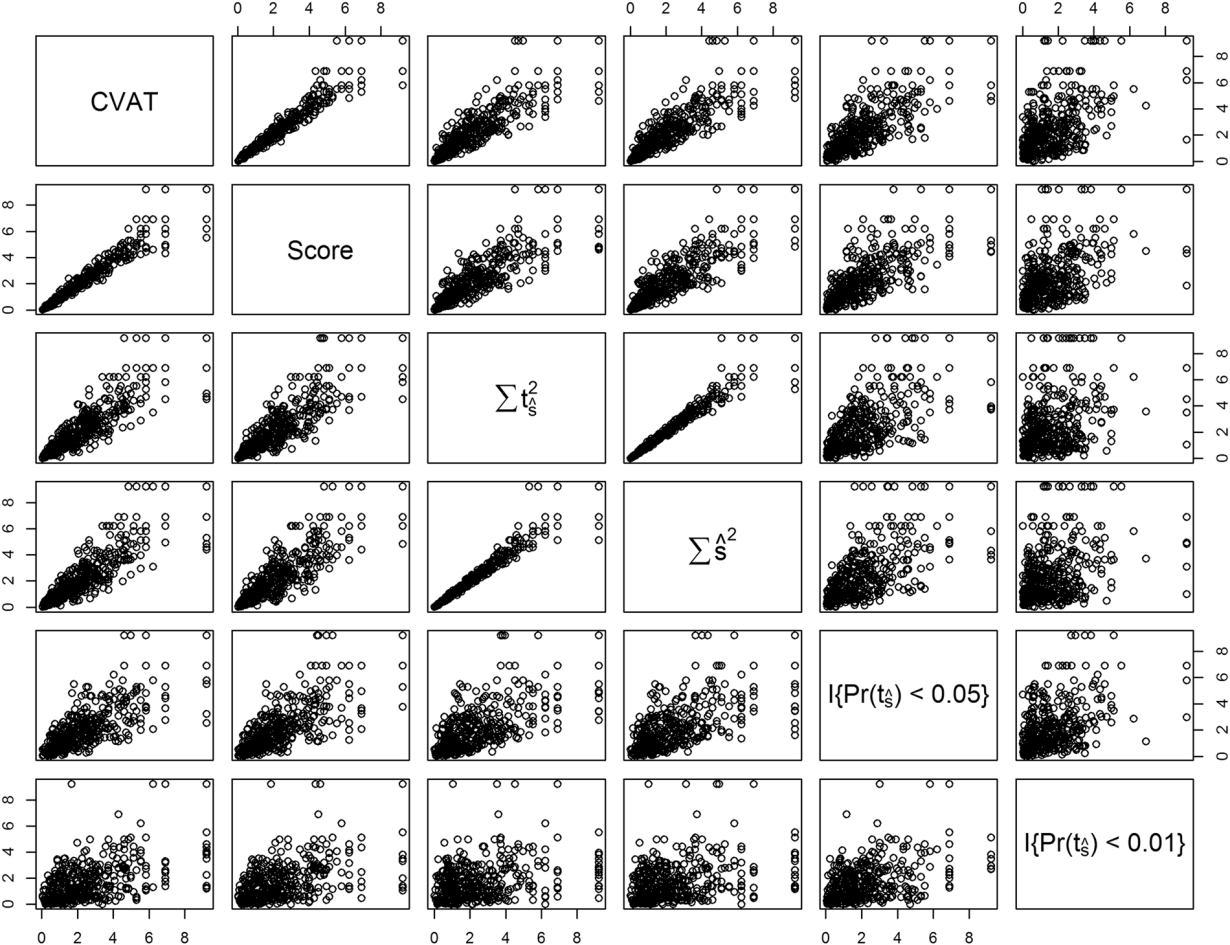
Relationship between the significance levels of different set test statistics. Scatter plots of all pairwise combinations of significance between the set test statistics, i.e. T_CVAT_ (CVAT), T_Score_ (Score), T_Sum_ using single marker effects $({\sum \widehat{{\rm{s}}}}^{2})$ or using single marker t-statistics $(\sum {{\rm{t}}}_{\widehat{{\rm{s}}}}^{2})$, and T_Count_ with a threshold p-value < 0.05 $({\rm{I}}\{{\rm{\Pr }}({{\rm{t}}}_{\hat{{\rm{s}}}}) < 0.05\})$ and p-value < 0.01 $({\rm{I}}\{{\rm{\Pr }}({{\rm{t}}}_{\hat{{\rm{s}}}}) < 0.01\})$. Significance, shown as −log(p), was measured for the association of simulated phenotype with genomic feature over a dilution range of adding 0 to 2000 non-causal SNPs to the *C*_1_ causal set. Plots are arranged such that all plots in a row share a common y-axis, and all plots in a column share a common x-axis. The names of the x- and y-axes are shown in the diagonal boxes. Genomic heritability was set to 30% (h^2^ = 0.3), and the proportion of genomic variance explained by the feature was 30% (${{\rm{h}}}_{{\rm{f}}}^{2}$ = 0.3). The random causal model was used, randomly selecting causal SNPs (*C*_1_ and *C*_2_) from the complete set of SNPs. Five replicates were used within each line (N_rep_ = 5).

The p-values of set test statistics T_CVAT_ and T_Score_ were highly correlated (0.96) with each other (Fig. 2). The figure shows the relationship between the minus logarithm of the p-values for the observed set test statistics. The results represented is for a genomic heritability of 30% and where the genomic feature explains 30% of the genomic variance (i.e. h^2^ = 0.3 and ${{\rm{h}}}_{{\rm{f}}}^{2}$ = 0.3). Although less pronounced, T_CVAT_ and T_Score_ also showed a high correlation with T_Sum_ of single marker effects $\widehat{{\rm{s}}}$ (0.87 and 0.85, respectively) and t-statistics (0.87 and 0.85, respectively). Lower correlations were observed between T_CVAT_ and T_Count_ at p-value cut-offs of 0.05 (0.76) and 0.01 (0.52). This was also the case for T_Score_ and T_Count_ showing a correlation of 0.74 at a p-value cut-off of 0.05 and 0.48 at p-values less than 0.01.

#### Relationship between set test statistics and predictive ability of the GFBLUP model

**Figure 3:**
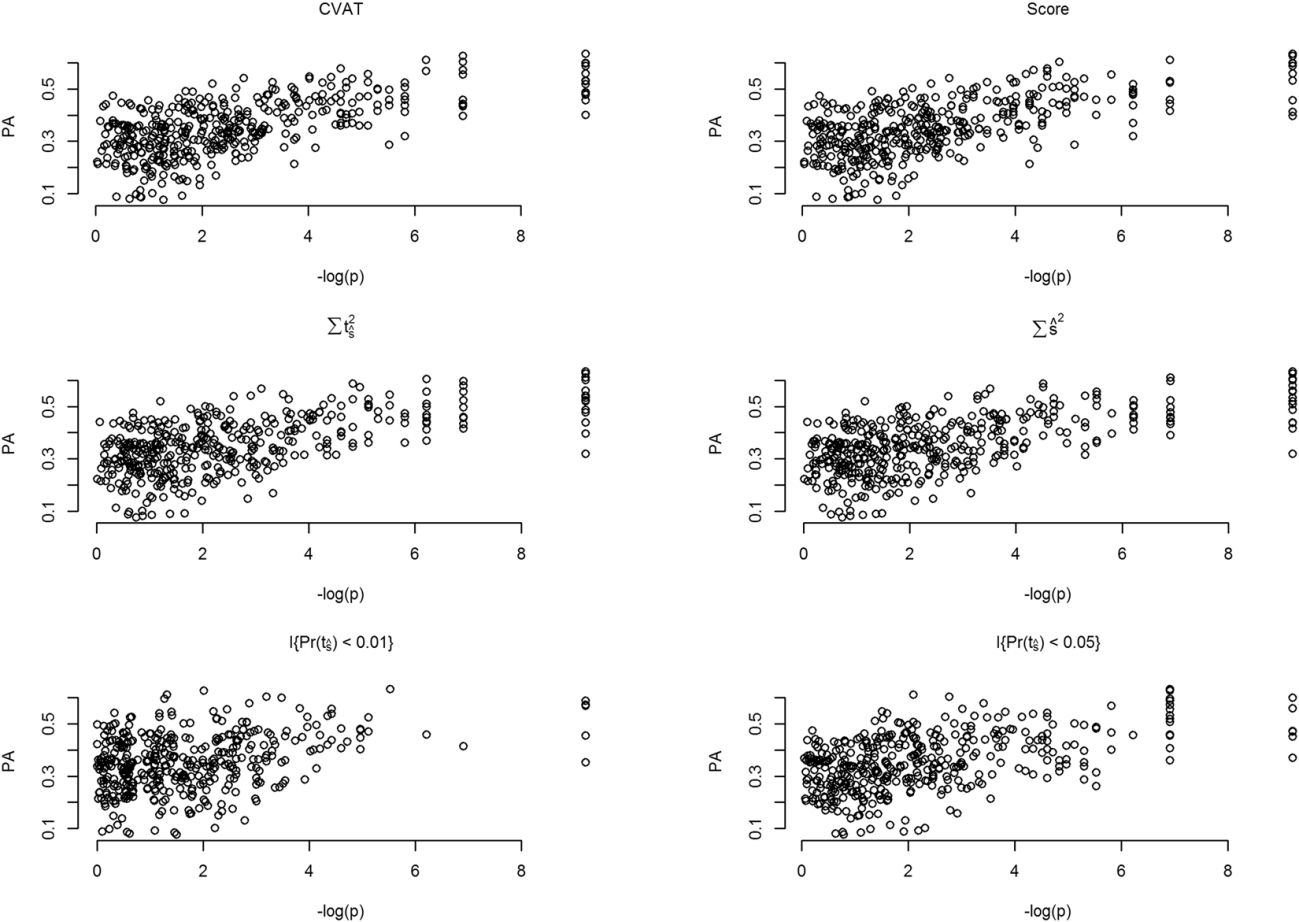
Relationship between the predictive ability of GFBLUP and significance levels of different set test statistics. Scatter plots showing the relationship between significance of set test statistics (x-axis) and predictive ability (PA, y-axis) of GFBLUP. Significance is expressed as −log(p). The different panels show results for the different set test statistics: T_CVAT_ (CVAT), T_Score_ (Score), T_Sum_ using single marker effects $({\sum \widehat{{\rm{s}}}}^{2})$ or using single marker t-statistics $(\sum {{\rm{t}}}_{\widehat{{\rm{s}}}}^{2})$, and T_Count_ with a threshold p-value < 0.05 $({\rm{I}}\{{\rm{\Pr }}({{\rm{t}}}_{\hat{{\rm{s}}}}) < 0.05\})$ and p-value < 0.01 $({\rm{I}}\{{\rm{\Pr }}({{\rm{t}}}_{\hat{{\rm{s}}}}) < 0.01\})$ Genomic heritability was set to 50% (h^2^ = 0.5), and the proportion of genomic variance explained by the feature was 30% (${{\rm{h}}}_{{\rm{f}}}^{2}$ = 0.3). The random causal model was used, randomly selecting causal SNPs (*C*_1_ and *C*_2_) from the complete set of SNPs. Five replicates were used within each line (N_rep_ = 5).

The three set test statistics (T_CVAT_, T_Score_, and T_Sum_) were all highly correlated to the predictive ability of the GFBLUP model (ranging from 0.59 to 0.62, respectively, Fig. 3). The pair-wise plots presented in Fig. 3 show the relationship between the minus logarithm of the p-value for the observed set test statistic and the predictive ability of the GFBLUP model. The correlation between predictive ability and the count based set test statistics was slightly lower ranging from 0.34 to 0.48.

### Influence of genomic feature and trait specific factors on detection power

**Figure 4:**
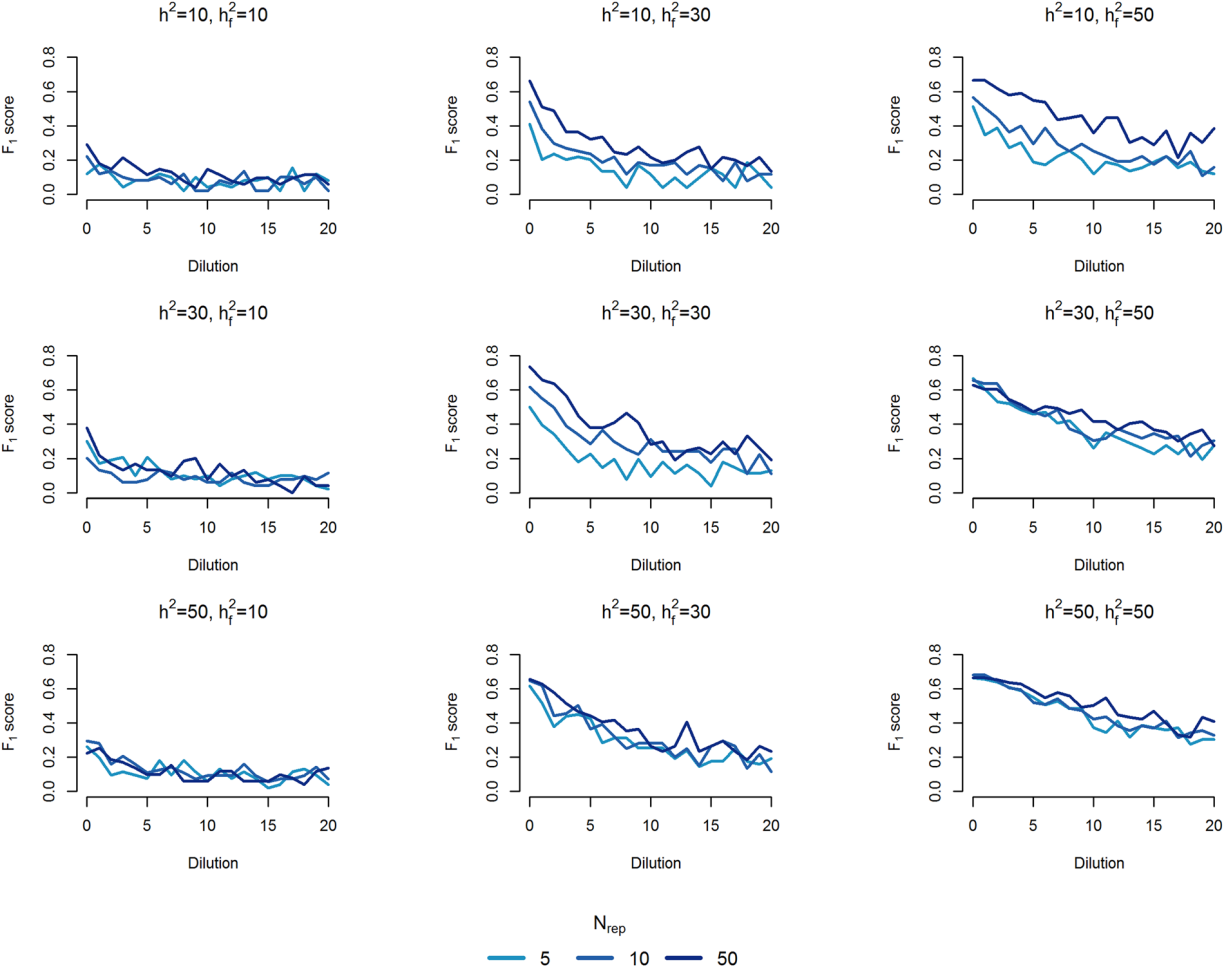
Influence of genomic feature, trait specific factors and dilution on detection power. In each row the heritability (h^2^) is kept constant while the proportion of genomic variance explained by the feature increases (${{\rm{h}}}_{{\rm{f}}}^{2}$ = 0.1, 0.2, 0.3, 0.5). Moving down each column h^2^ increases from 0.1 to 0.2 and 0.5 while ${{\rm{h}}}_{{\rm{f}}}^{2}$ is kept constant. The power to detect features enriched for causal variants was quantified by the F_1_ score shown on the y-axis of each panel. P-value cut-off for the set test statistic was 0.05. F_1_ score is shown as a function of dilution, i.e. adding up to 2000 non-causal SNPs to the feature, on the x-axis. The number of replicates (N_rep_ = 5, 10 and 50) within line is depicted by the colour scale.

Here we present the results for the GBLUP-derived CVAT set test statistic (Fig. 4). We focus on the results of the CVAT test statistic since it had the best performance (i.e. highest F_1_ score across all simulation scenarios, Fig. 1). The patterns observed are very similar for the other set test statistics (results not shown). Power to detect genomic features affecting the phenotypes was influenced both by trait and genomic feature specific factors. The proportion of the genomic variance explained by the genomic feature (${{\rm{h}}}_{{\rm{f}}}^{2}$) greatly impacted detection power (higher levels of power from left to right columns of Fig. 4) and robustness towards dilution, i.e. increasing the proportion of non-causal SNPs in the genomic feature (curves as a function of dilution are more steep with decreasing ${{\rm{h}}}_{{\rm{f}}}^{2}$ in Fig. 4). Power to detect genomic features was low if both genomic heritability and proportion of genomic variance explained by genomic feature was low (${{\rm{h}}}_{{\rm{f}}}^{2}$ = 0.1 and h^2^ = 0.1), even without dilution. Impact of dilution was less severe when the proportion of genomic variance explained by genomic feature was highest (${{\rm{h}}}_{{\rm{f}}}^{2}$ = 0.5). This increased robustness towards dilution resulted in power above 40% in all cluster model scenarios with N_rep_ = 50 replicates within line and a genomic heritability of 50%. The level of genomic heritability (h^2^) was positively correlated with power (Fig. 4). However, at high ${{\rm{h}}}_{{\rm{f}}}^{2}$ and in absence of dilution all genomic features were detected regardless of overall genomic heritability, but with some false positives. Furthermore, if ${{\rm{h}}}_{{\rm{f}}}^{2}$ was high, the detection power of CVAT for high heritability traits were less affected by dilution than low heritability traits (steeper slope of upper-right panel, compared to lower-right panel of Fig. 4). Dilution decreased power in all simulation scenarios (decreasing curves on all panels of Fig. 4). Detection power was slightly higher if causal SNPs in the genomic feature were clustered in smaller regions as compared to distributed randomly on the genome (results not shown). Furthermore, detection power increases with increasing numbers of replicates within line (N_rep_ = 5, 10, or 50).

### Application of CVAT on CCRT data

Since the simulation study suggested that CVAT was the most powerful set test statistic, we applied CVAT and its extensions to CCRT data.

#### Determination of linear mixed model to be used for CVAT analysis

Initially we fitted a series of linear mixed models (GBLUP or GFBLUP) to determine the final model to be used in the subsequent CVAT analyses of the CCRT trait in DGRP. Models were fitted for single and multiple traits, including one or two features (in this case genes and inter-genic regions) and considering gene based and genome based null hypotheses. Trait heritability for CCRT estimated using a multiple trait GBLUP model was 0.42 for males and 0.48 for females. The genetic correlation between males and females was 0.97. Partitioning genomic variance into genes and inter-genic regions, using a two-component genomic feature model, did not significantly improve the model fit (likelihood ratio test statistic less than one; p-value > 0.1). The empirical distribution of the CVAT test statistic was determined under two null hypotheses: One involving random sampling of genetic markers from gene regions (gene based), and one based on random sampling of genetic markers from the whole genome (whole genome based). Under the gene based null hypothesis GO terms were slightly more significant compared to the whole genome based null hypothesis both in the case of females and males (Fig. S1 panels (**a**) and (**b**) respectively). Furthermore, the association of GO terms with CCRT was highly correlated between males and females (Fig. S1 panel (**c**) and (**d**)) under both the gene based or whole genome null hypothesis (correlation = 0.95 and 0.98 respectively).

The significance of T_CVAT_ did not show a considerable difference whether using the two trait or single trait models (the third extension to T_CVAT_, Fig. S1 panel (**e**) and (**f**)). Therefore, results for the CCRT trait reported here are from a one-component, two trait model using a gene based null hypothesis.

#### CCRT associated GO terms and genes detected using CVAT

Several GO terms were significantly associated to CCRT in both males and females (p-value adjusted for multiple tests ≤0.001, Table 1). Table 1 shows the highest-ranking GO terms for males and females (MT-CVAT within trait), as well as the significance of GO terms when considering the covariance between the total genomic effect for *all markers* in males, and the genomic effect for *markers* in the feature for females and vice versa (MT-CVAT across traits). Eight GO terms for females and nine GO terms for males were significantly associated with CCRT. Males and females shared all but one of the most significant GO terms (p-value ≤ 0.001). The GO term “ATP-dependent DNA helicase activity” (GO:0004003, p = 0.0015) for females being only slightly above the 0.001 p-value cut-off. The *across trait* (traits being females and males) MT-CVAT set test results showed similar patterns as the *within trait* CVAT set test.

**Table 1:** Gene ontology terms significantly associated with CCRT for males and females.

GO id^a^	Empirical p-values^b,c,d^				Ontology^e^	Gene Ontology term
	Female	Male	Male/Female covariance	Female/Male covariance		
GO:0007266	<1 × 10^−4^	<1 × 10^−4^	<1 × 10^−4^	<1 × 10^−4^	BP	Rho protein signal transduction
GO:0035160	<1 × 10^−4^	<1 × 10^−4^	<1 × 10^−4^	<1 × 10^−4^	BP	Maintenance of epithelial integrity, open tracheal system
GO:0005100	<1 × 10^−4^	<1 × 10^−4^	<1 × 10^−4^	<1 × 10^−4^	MF	Rho GTPase activator activity
GO:0016323	2 × 10^−4^	3 × 10^−4^	1 × 10^−4^	4 × 10^−4^	CC	Basolateral plasma membrane
GO:0007173	3 × 10^−4^	7 × 10^−4^	7 × 10^−4^	6 × 10^−4^	BP	Epidermal growth factor receptor signaling pathway
GO:0035277	3 × 10^−4^	<1 × 10^−4^	<1 × 10^−4^	3 × 10^−4^	BP	Spiracle morphogenesis, open tracheal system
GO:0008289	5 × 10^−4^	4 × 10^−4^	3 × 10^−4^	4 × 10^−4^	MF	Lipid binding
GO:0007494	1 × 10^−3^	6 × 10^−4^	4 × 10^−4^	2 × 10^−4^	BP	Midgut development
GO:0004003	1.5 × 10^−3^	2 × 10^−4^	5 × 10^−4^	8 × 10^−4^	MF	ATP-dependent DNA helicase activity

^a^GO id = gene ontology id. ^b^p-values smaller than or equal to 0.001 were included for each sex. ^c^The empirical distributions were obtained by randomly sampling from gene regions on the genome. ^d^10,000 permutations were performed for each GO term. For p-values less than 1 × 10^−4^, more permutations would yield more precise p-values. ^e^BP = Biological Process, MF = Molecular Function and CC = Cellular Component.

**Figure 5:**
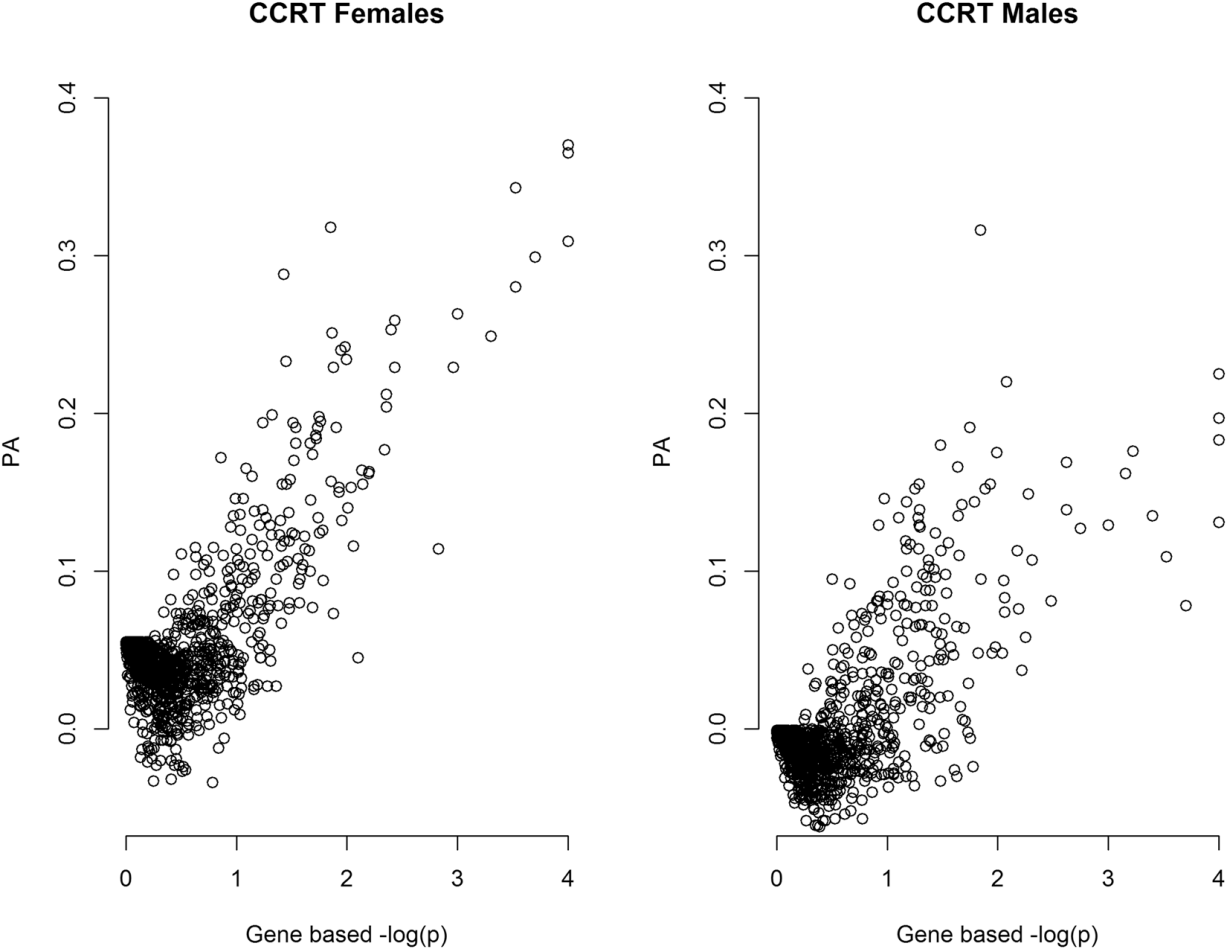
Relationship between gene ontology (GO) term CVAT test statistic and predictive ability of the GFBLUP model for chill coma recovery time (CCRT). The significance of GO terms related to CCRT in *Drosophila melanogaster* in females and males as determined by the CVAT test statistic (expressed as gene based −log(p), x-axis) from single trait analyses, plotted against the predictive ability (PA) of the single trait GFBLUP model (y-axis).

However, all GO terms reported in Table 1 had unadjusted p-values below 0.01, suggesting that these may be biologically relevant for CCRT in both sexes. In addition, the top-ranking GO terms significantly associated with CCRT were also predictive of the phenotypes as assessed in a cross validation study (Fig. 5).

**Figure 6:**
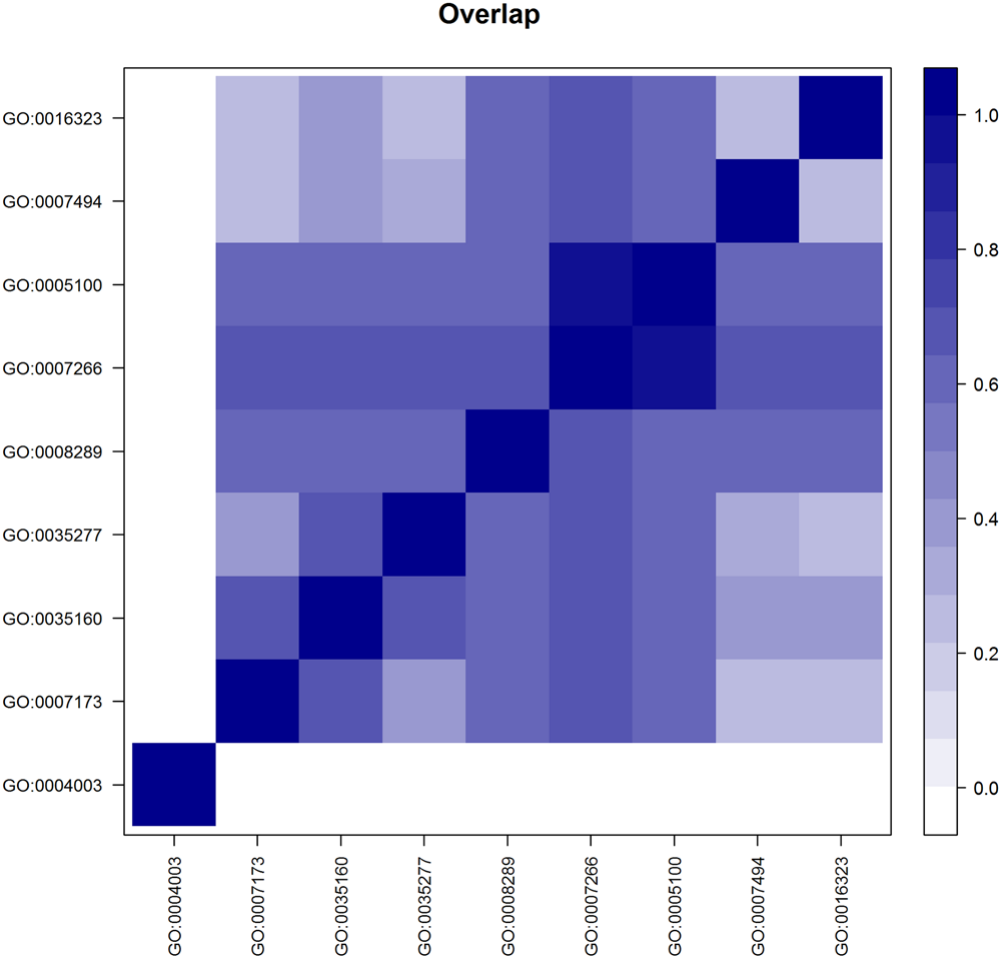
Heatmap showing the overlap between SNPs of significant GO terms. Each square [*i*, *j*] shows the proportion of SNPs associated with GO term *i*, as well as GO term *j*. Where *i* indexes rows and *j* indexes columns. Darker colours represent larger proportions of SNPs that overlap between GO terms. Only the most significant GO terms, presented in Table 1, are included.

There was a substantial overlap among the SNPs associated with each of the GO terms (Fig. 6). In particular, “Rho GTPase activator activity” (GO:0005100) and “Rho protein signal transduction” (GO:0007266) shared more than 98% of the SNPs. These two GO terms also shared a substantial number of SNPs (59–67%) with the remaining GO terms except for “ATP-dependent DNA helicase activity” (GO:0004003) which did not share any SNPs with the other GO terms.

**Figure 7:**
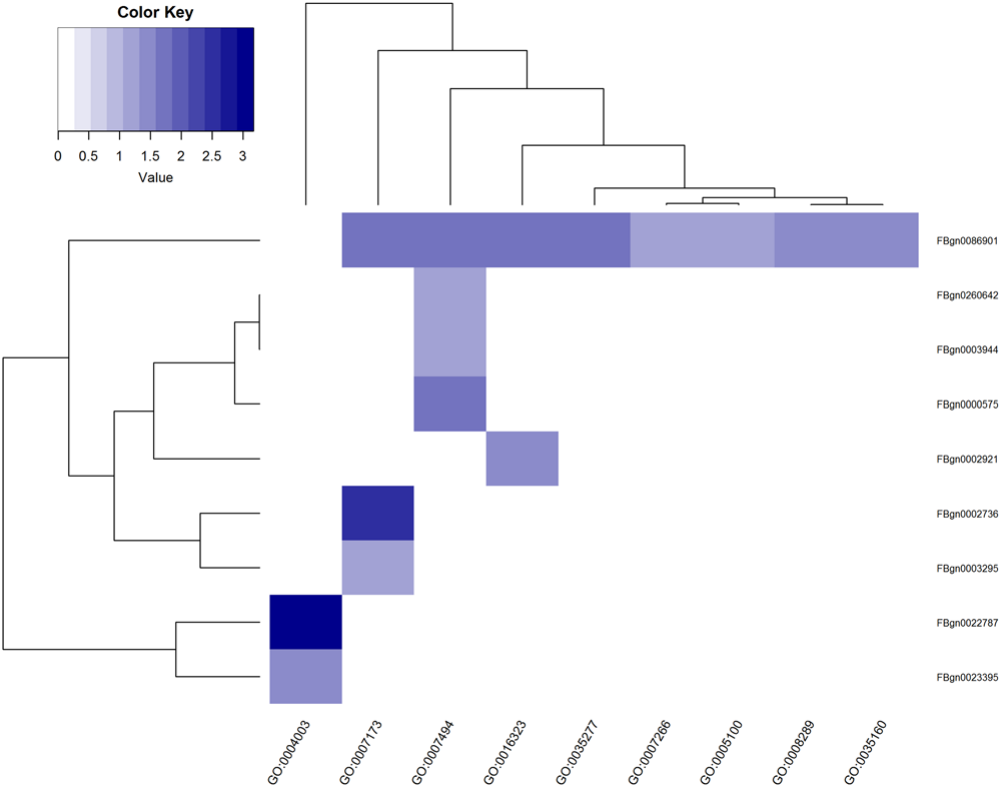
Heatmap showing the individual genes associated to CCRT for each of the top GO terms. The colour scale indicates the degree of association (expressed as −log(p)). The darkest blue colour indicates −log(p) = 4 and as the colour fades p-values increase.

Considering the overlap of SNPs between GO terms, further investigations were required to better understand the biological relevance of the CVAT results obtained at the GO term level. Therefore, we applied the CVAT set test at the individual gene level using only the genes that were part of the significantly associated GO terms. This enabled us to identify a number of genes that were associated with CCRT (Fig. 7). In particular, we found that *RhoGAP88C* (FBgn0086901) was significantly associated with CCRT and that this gene is part of all but one of the significant GO terms (Fig. 7). In addition, we found evidence that several other genes including *antennapedia* (FBgn0260642), *ultrabithorax* (FBgn0003944), and *extra macrochaetae* (FBgn0000575) contributed to the significance of the GO term “Midgut development” (GO:0007494), and the genes *mago nashi* (FBgn0002736) and *roughoid* (FBgn0003295) contributed to the significance of the GO term “Epidermal growth factor receptor signaling pathway” (GO:0007173). Finally, we found that the genes *Chd3* (FBgn0023395) and *helicase 89B* (FBgn0022787) contributed to the significance of the GO term “ATP-dependent DNA helicase activity” (GO:0004003).

## Discussion

We demonstrated that GBLUP-derived set tests are powerful for detecting genomic features enriched for causal variants affecting a quantitative trait in populations with a low degree of linkage disequilibrium. The different set tests were compared using simulated data generated from DGRP genotypes further illustrating the impact of trait- and genomic feature-specific factors on detection power. These set tests provide a formal statistical modeling framework for borrowing and evaluating information across a wide range of experimental studies that may help provide novel insights into genetic and biological mechanisms underlying complex traits. The methods are computationally fast allowing us to rapidly analyze many different classes of genomic features. This will help to discover genomic features enriched for causal variants that can be used to develop more accurate predictions using GFBLUP models. GBLUP-derived set tests are based on a flexible linear mixed modelling framework that allows us to adjust for other known genetic and non-genetic factors, while using existing standard software. Importantly, the GBLUP models can be extended in several ways that potentially can increase detection power.

### Comparison of set tests

Several GBLUP-derived set tests were compared in terms of statistical power to detect genomic features enriched for causal variants. Despite GBLUP being considered a “black box” modeling approach we showed that it is possible to derive powerful set tests from it. In particular, in all scenarios evaluated we showed that the covariance association test (CVAT) had similar power to a commonly used score based approach^28^ (also known as the sequence kernel association test, SKAT), and that both CVAT and SKAT outperformed the methods based on summing the number of single marker statistics in the feature. Both CVAT and SKAT are fast and powerful methods to identify genomic features enriched for causal variants and thereby contribute to develop more accurate prediction models. One advantage of the CVAT approach is that it builds on a flexible linear mixed modelling framework that can be extended in several ways that potentially can increase detection power. Extensions include the consideration of different levels of a hierarchical feature, multiple genetic components having different genomic value distributions and a multiple trait GBLUP.

Set tests based on counting test statistics (T_Count_) appear to have lower power compared to test statistics based on summing the squared single marker statistics (T_Sum_). This may in part be explained by the simulated genetic architecture which were enriched for causal variants with small to moderate effects. In general, methods based on a count test statistic are likely to have high power to detect association if the genomic feature harbours genetic markers with large effects, but it will not detect a genomic feature with many genetic markers having small to moderate effects^45^. Our results show that in such cases, it is more powerful to use a test statistic, such as the mean or sum of the single marker statistics for the genomic feature.

Finally, we have shown a clear link between the significance levels of the set test statistics and the level of predictive ability using these sets as features in the GFBLUP model. This link could be exploited to build more accurate GFBLUP models in a computationally efficient way. That is, using the GBLUP model to identify genomic features enriched for associated variants and subsequently apply the identified sets as features in the GFBLUP model.

### Factors influencing detection power

Several trait and feature specific factors can influence the power to determine whether a genomic feature is enriched for causal variants. Power is positively correlated with the proportion of genomic variance explained by the genomic feature, and power decreases with the addition of non-causal SNPs in the feature (dilution). Furthermore, the genetic architecture of the causal variants (distributed randomly or clustered along the genome) also influenced power. The increased detection power and resistance towards dilution in the case where the true causal SNPs are clustered in smaller genomic regions is likely due to larger effect size of individual markers in these regions. Not surprisingly, power is increased if the trait is highly heritable and the number of phenotypic records available is high. These patterns were consistent across the different set tests and are factors that need to be considered in the analyses of real data.

#### Influence of linkage disequilibrium on detection power

We compared the GBLUP derived set tests based on genotypes obtained from the sequenced inbred lines of the *Drosophila melanogaster* Genetic Reference Panel. The population consist of 205 largely unrelated lines with a low degree of linkage disequlibrium across their genomes. Thus, our results suggest that GBLUP-derived set tests may have high power in situations where individuals are largely unrelated such as human study populations. In a population of highly related individuals the general genomic relationship will be a good approximation of the genomic relationship at the true causal variants^2^. This will lead to more accurate estimates of overall genomic value. On the other hand due to extensive linkage disequilibrium it may be difficult to accurately estimate single marker effects and this will in turn influence the feature set test statistic. Therefore more research is required to understand the influence of genetic relatedness and degree of linkage disequilibrium on detection power of the GBLUP derived set tests.

#### Influence of null hypothesis on detection power

In this study we compared the set tests using a competitive null hypothesis. The competitive null hypothesis states that the degree of association within a genomic feature is equal to that of a random set of genetic markers. An alternative is the *self*-*contained* null hypothesis^46–48^. The self-contained null hypothesis states that the genomic feature, by it self, does not display any association to the phenotypic trait. This is usually done by testing whether the variance component or the test statistics for the genomic feature are zero. The self-contained may be preferable over a competitive, as it has more power in general^46^, and the interpretation is simpler, as it determines whether there is association or not. On the other hand the competitive null hypothesis is perhaps more biologically relevant as it is in agreement with the infinitesimal model^49, 50^ which is a commonly used genetic model assuming that there are many causal variants each with small to moderate effects underlying the complex trait.

### Further extensions of GBLUP-derived set tests and alternative methods

The GBLUP-derived set test modeling framework can be extended in several ways that potentially can increase detection power. First, multiple feature sets can be fitted in the model (e.g. a GFBLUP model), such as grouping markers based on their minor allele frequency^19, 20^ or prior QTL information^16^. By fitting multiple feature sets genetic effects are estimated based on a mixture of normal distributions enabling further differential shrinkage of single marker effects across feature sets. Second, further shrinkage of single marker effects within features may be achieved by using a weighted genomic relationship matrix^11, 51^ for each feature set. Third, a multiple trait GBLUP model^21, 22^ can be fitted. This can increase the accuracy of the overall genomic effect^21, 22^ and thereby the single marker effect which in turn will lead to a more accurate test statistic for the genetic marker set. Fourth, in animal and plant populations with extended pedigrees we might use information on inviduals without genotype information^51^ to increase accuracy of the overall genomic value. We are currently investigating these extensions hypothesizing that they, in some situations, may lead to increased power of the GBLUP-derived set test.

#### Comparison to Bayesian methods

The GBLUP and GFBLUP models used in this study can also be implemented using Bayesian methods^52–55^. In particular Bayesian mixture models such as BayesB^56^, BayesR^56, 57^ or Bayesian Lasso models^58^ are relevant alternative methods. For these methods it is also possible to derive test statistics that quantify the joint effect of the markers in the feature set. Furthermore, they also allow for differential shrinkage of marker effects within feature sets and can be used to fit multiple feature sets. More investigations are required to compare these methods to the GBLUP-derived set test and investigate to what extend these methods will increase detection power.

### Application of CVAT on CCRT

Although the main objective of this paper was to compare different GBLUP derived set tests, we would like to discuss, albeit very condensed, the plausible biological relevance of our results.

CCRT was strongly associated with the GO terms ‘Rho protein signal transduction’ (GO:0007266) and ‘Rho GTPase activator activity’ (GO:0005100) in both males and females. Rho genes’ functional relevance, with regards to CCRT, has been implied by their involvement in (a) intracellular signal transduction pathways^59^, (b) indirectly mediating circadian rhythm, through actin regulation^60^ as well as (c) contributing to ion homeostasis by regulating K^+^ channel cell surface expression^61^.

“Midgut development” (GO:0007494) was also among the high-ranking GO terms for association with CCRT. It is well established that the midgut of insects is an important site for the exchange of ions with the hemolymph^62, 63^. Insect cold resistance is directly related to maintenance of water and ion homeostasis^64–66^. In the fall field cricket, *Gryllus pennsylvanicus*, the midgut has been shown to be the most sensitive site for the exchange of ions and water during cold exposure^67^. In the midgut cold exposure caused rising Na^+^ levels causing a disruption in water homeostasis ultimately leading to an increased K^+^concentration in the hemolymph^65^. It is ultimately this increased K^+^ concentration that causes an electrophysiological failure of the neuromuscular system and subsequent chill-coma^68–70^.

There was a substantial overlap among the SNPs associated with each of the top-ranking GO terms. In order to zoom in on relevant genes underlying these GO terms, the CVAT set test was also applied at the individual gene level of the significant GO terms. This enabled us to identify a number of the genes that was associated to CCRT. In particular, we found that the *crossveinless*-*c* gene (FBgn0086901), was highly significant and is a part of both Rho protein signal transduction and Rho GTPase activator activity GO terms^42^. *Crossveinless*-*c* is an important regulator of Rho GTPase activity^71^. The Rho-family of GTPases are in turn associated with the direct regulation of the actin cytoskeleton^72^. Chilling has been shown to disrupt cytoskeletal organization in primary embryonic cultures of *Drosophila* cells^73^. Interestingly, diapausing mosquitos (*Culex pipens*) have greater abundance of polymerized actin at muscle fiber intersections in the midgut^74^. Thus, regulation of cytoskeletal function may be implicated as an important component of cold acclimation.

In general, biological interpretation might be hampered by the definition (or misspecification) of the genomic feature and a potential large overlap in the genetic marker sets between the different genomic feature classes. In the latter case, biological interpretation may be improved by using methods that take the overlap into account^75^.

## Conclusion

GBLUP-derived set tests are powerful compared to existing methods for detecting genomic features enriched for causal variants in populations with a low degree of linkage disequilibrium. The tests can be implemented using standard BLUP models, and can be extended in several ways that potentially can increase detection power. The methods are computationally fast allowing us to rapidly analyze many different classes of genomic features. This will help to discover genomic features enriched for causal variants that can be used to develop more accurate predictions using GFBLUP models.

## Electronic supplementary material

Supplementary Figure 1
