# Supplementary material for: Multiple Trait Covariance Association Test Identifies Gene Ontology Categories Associated with Chill Coma Recovery Time in Drosophila melanogaster
Source: Sci Rep. 2017 May 25;7:2413. doi: 10.1038/s41598-017-02281-3 (PMC5445101; doi:10.1038/s41598-017-02281-3)
Supplement: Supplementary file 1 — Supplementary Figure 1 [file 41598_2017_2281_MOESM1_ESM.pdf]

Multiple Trait Covariance Association Test Identifies Gene Ontology Categories Associated to Chill Coma Recovery Time in *Drosophila melanogaster*

Izel F. Sørensen, Stefan M. Edwards, Palle Duun Rohde, Peter Sørensen

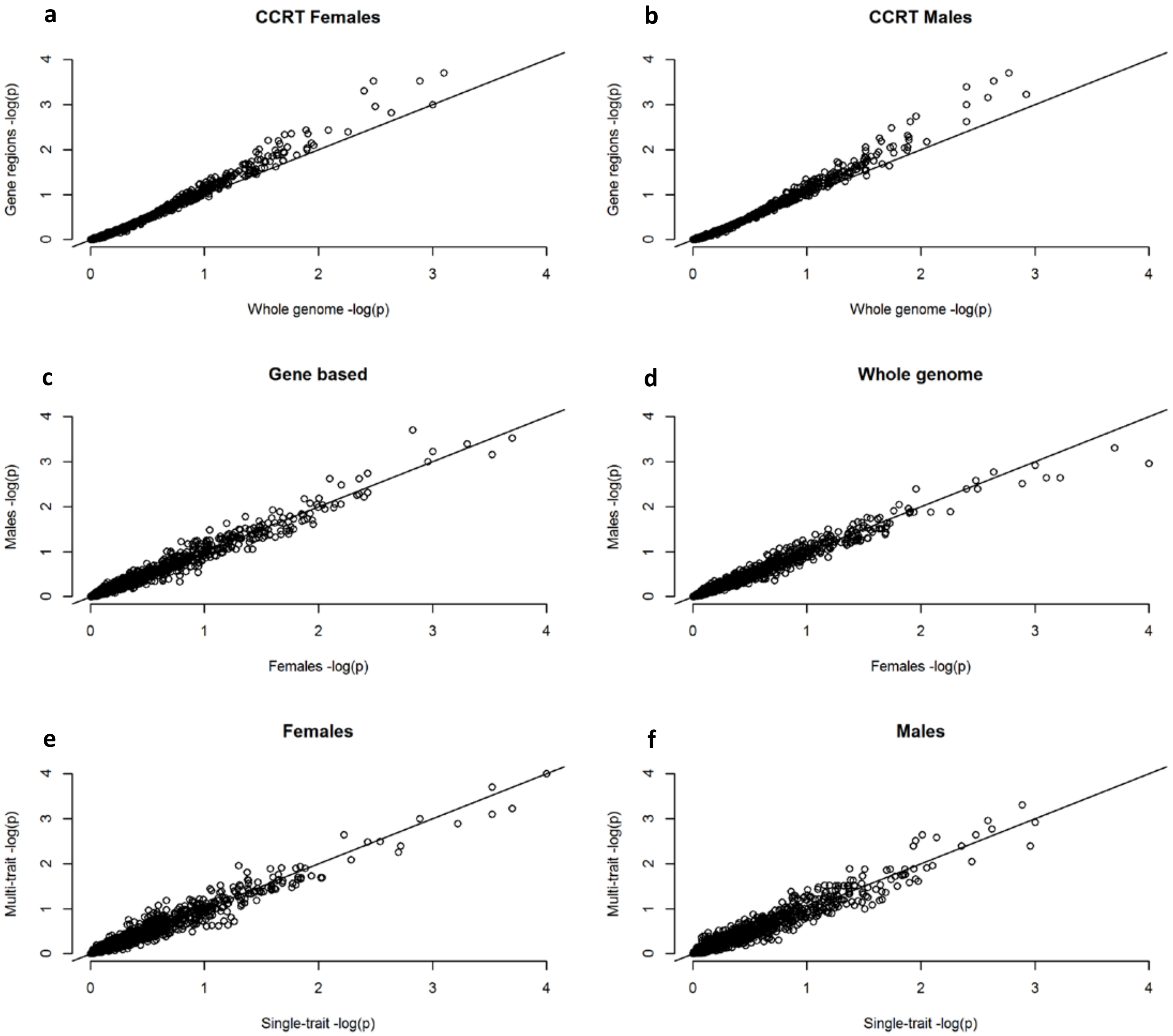

Figure S1. Correlation between the significance of CVAT statistics for chill coma recovery time (CCRT) under different scenarios of the mixed model. Panels (**a**) and (**b**) show the correlation between the significance of T_{CVAT} for GO terms (expressed as -log(p)) under the whole genome based null hypothesis and the gene based null hypothesis for females and males respectively. Gene based -log(p) for males and females were highly correlated (panel (**c**)), as were whole genome based -log(p) (panel (**d**)). Relationships between the gene based -log(p) using a single trait or multiple trait model are depicted in panels (**e**) and (**f**) for females and males respectively . The figure displays the significance of 1117 test statistics (for 1117 GO terms).
